# The Need for Establishing a Universal CTG Sizing Method in Myotonic Dystrophy Type 1

**DOI:** 10.3390/genes11070757

**Published:** 2020-07-07

**Authors:** Alfonsina Ballester-Lopez, Ian Linares-Pardo, Emma Koehorst, Judit Núñez-Manchón, Guillem Pintos-Morell, Jaume Coll-Cantí, Miriam Almendrote, Giuseppe Lucente, Andrea Arbex, Jonathan J. Magaña, Nadia M. Murillo-Melo, Alejandro Lucia, Darren G. Monckton, Sarah A. Cumming, Alba Ramos-Fransi, Alicia Martínez-Piñeiro, Gisela Nogales-Gadea

**Affiliations:** 1 Neuromuscular and Neuropediatric Research Group, Institut d’Investigació en Ciències de la Salut Germans Trias i Pujol, Campus Can Ruti, Universitat Autònoma de Barcelona, 08916 Badalona, Barcelona, Spain; aballester@igtp.cat (A.B.-L.); ilinares@igtp.cat (I.L.-P.); ekoehorst@igtp.cat (E.K.); judith3194@gmail.com (J.N.-M.); guillempintos@gmail.com (G.P.-M.); jcollc2@gmail.com (J.C.-C.); miriam.almendrote@gmail.com (M.A.); glucente@igtp.cat (G.L.); andreaarbex@gmail.com (A.A.); aramosfransi@gmail.com (A.R.-F.); aliwonpi@gmail.com (A.M.-P.); 2 Centre for Biomedical Network Research on Rare Diseases (CIBERER), Instituto de Salud Carlos III, 28029 Madrid, Spain; 3 Division of Rare Diseases, Vall d’Hebron University Hospital, 08035 Barcelona, Spain; 4 Neuromuscular disorders Unit. Neurology Service. Neuroscience department, Hospital Universitari Germans Trias i Pujol, 08916 Badalona, Barcelona, Spain; 5 Hospital Municipal de Badalona, 08911 Badalona, Barcelona, Spain; 6 Laboratory of Genomic Medicine, Department of Genetics, National Rehabilitation Institute (INR-LGII), 14389 Mexico City, Mexico; maganasm@hotmail.com (J.J.M.); phoenicopterix_ruber@hotmail.com (N.M.M.-M.); 7 Faculty of Sport Sciences, Madrid, Spain, & Instituto de Investigación Hospital 12 de Octubre (imas12), Universidad Europea, 28041 Madrid, Spain; alejandro.lucia@universidadeuropea.es; 8 Institute of Molecular, Cell and Systems Biology, College of Medical, Veterinary and Life Sciences, University of Glasgow, Glasgow G128QQ, UK; Darren.Monckton@glasgow.ac.uk (D.G.M.); sarah.cumming@glasgow.ac.uk (S.A.C.)

**Keywords:** CTG expansion size, myotonic dystrophy type 1, long PCR, small pool-PCR, age of disease onset

## Abstract

The number of cytosine-thymine-guanine (CTG) repeats (‘CTG expansion size’) in the 3′untranslated region (UTR) region of the *dystrophia myotonica*-protein kinase (*DMPK*) gene is a hallmark of myotonic dystrophy type 1 (DM1), which has been related to age of disease onset and clinical severity. However, accurate determination of CTG expansion size is challenging due to its characteristic instability. We compared five different approaches (heat pulse extension polymerase chain reaction [PCR], long PCR-Southern blot [with three different primers sets—1, 2 and 3] and small pool [SP]-PCR) to estimate CTG expansion size in the progenitor allele as well as the most abundant CTG expansion size, in 15 patients with DM1. Our results indicated variability between the methods (although we found no overall differences between long PCR 1 and 2 and SP-PCR, respectively). While keeping in mind the limited sample size of our patient cohort, SP-PCR appeared as the most suitable technique, with an inverse significant correlation found between CTG expansion size of the progenitor allele, as determined by this method, and age of disease onset (r = −0.734, *p* = 0.016). Yet, in light of the variability of the results obtained with the different methods, we propose that an international agreement is needed to determine which is the most suitable method for assessing CTG expansion size in DM1.

## 1. Introduction

Myotonic dystrophy type 1 (DM1) is a multisystemic disorder with an autosomal dominant inheritance pattern. DM1 is caused by unstable expansion of CTG repeats in the 3′ untranslated (UTR) region of the *dystrophia myotonica*-protein kinase (*DMPK*) gene [1]. Non-affected individuals usually have 5 to 35 CTG repeats, with carriage of 35 to 49 repeats, leading to a “pre-mutational” phenotype that is not usually associated with clinical manifestations. In turn, patients with DM1 typically have more than 50 CTG repeats in the blood, and sometimes up to several thousands [2]. Furthermore, because CTG expansion is highly unstable and prone to increase in these patients, an eventual decrease (or ‘contraction’) in the number of CTG repeats is typically biased toward further expansion in the context of DNA replication, recombination, transcription and/or repair [3,4,5,6,7]. Consequently, CTG expansion shows a characteristic somatic mosaicism [8].

Determination of the actual number of CTG repeats is complicated by the immense length of the expanded alleles as well as by the highly stable secondary structures that are created inside the repetitive guanine-cytocine (GC)-rich sequence. In addition, it is possible to underestimate rare mutant molecules in both germline and somatic cells. Accurate assessment of CTG expansion size is important in patients with DM1. Indeed, the number of CTG repeats can be inversely and directly related with age of disease onset and clinical severity, respectively [9,10]. Although different approaches have been described to assess CTG expansion size in patients with DM1 [5,11,12,13,14,15], some methodological issues remain to be solved, mainly related to the inherent repeat instability and technical difficulties when amplifying long CTG fragments.

It was therefore the purpose of this study to compare the number of CTG repeats (‘expansion size’) of the progenitor allele and the mode allele between five different assays using three different methods: (i) heat pulse extension (HPE)-polymerase chain reaction (PCR); (ii) long PCR (LPCR)1-Southern blot (SB); (iii) LPCR2-SB; (iv) LPCR 3-SB; and (v) small pool (SP)-PCR. The rationale behind this work was to determine whether the different methodologies that are currently available yield comparable results, so that it is necessary—or not—to come to a consensus as to which methodology should be used.

## 2. Materials and Methods

### 2.1. DNA Extraction and Subjects

This study was approved by the Ethics Committee of the University Hospital Germans Trias i Pujol (Badalona, Spain) and was performed in agreement with the Declaration of Helsinki for Human Research in 1975. All subjects signed a written informed consent to participate in the study. Total genomic DNA was obtained from peripheral blood in 15 patients with DM1 and 10 controls, as previously described [16]. Diagnosis of DM1 was confirmed by triplet primed-PCR, as reported elsewhere [17]. In controls, CTG number in both *DMPK* alleles was assessed by Sanger sequencing.

### 2.2. Heat Pulse Extension-PCR

First, we amplified the genomic DNA from patients and controls using HPE-PCR, as described elsewhere [13]. Unlike conventional PCR, in which the extension step is performed at a constant temperature, HPE-PCR includes multiple heat pulses in the extension step. Heat pulses temporarily destabilize the secondary structures formed in the long GC-rich repetitive sequence, thereby improving the extension efficiency and the amplification of the long expansions. The technique was carried out using the same PCR conditions, reagents, the equivalent taq polymerase (Phusion High-Fidelity DNA Polymerase, Thermo Fisher Scientific; Waltham, MA, USA) and the same thermocycler (GeneAmp 9700 thermal cycler, Applied Biosystems, Foster City, CA, USA) as the Orpana et al. study [13]. The sizing of the CTG expansion was performed in a 1% agarose gel using the molecular ladder NZYDNA Ladder III (NZYTech, Lisboa, Portugal).

### 2.3. Long PCR-Southern Blot

The DNA samples (100 ng per reaction) were amplified with three different primer sets in a LPCR: (i) DM102 and DM101 (LPCR1); (ii) MDY1D and SOMY4R (LPCR2); (iii) MDY1D and DM1rev (LPCR3). We used the LongAmp^®^ Taq PCR Kit (New England BioLabs, Ipswich, MA, USA) and the GeneAmp 9700 thermal cycler (Applied Biosystems, Foster City, CA, USA). The set conditions for each of the three LPCR methods were as follows: initial denaturation at 94 °C for 4 min, followed by 35 cycles of denaturation at 94 °C for 30 s, and annealing–extension at 65 °C for 7 min. Final extension was performed at 65 °C for 10 min. Subsequently, LPCR products were analyzed using SB. In brief, all LPCR products were electrophoresed in a 0.8% agarose gel at 80V for 90 min, and transferred to a nylon membrane (Roche; Basel, Switzerland) after gel washing with an acid solution (250 mM HCl) for 15 min, a basic solution (0.5M NaOH) for 30 min, and a neutralizing solution (0.5 M Tris-HCl, pH = 7.5, 1.5 M NaCl) for 30 min. DNA was fixed to the membrane by incubation for 1 h 15 min at 65 °C. We used a concentration of 10 pmol/mL DIG-labeled LNA probe (5′-gcAgCagcAgCagCagcAgca-3′, where capital letters indicate LNA nucleotides) to hybridize the membrane for 3 h at 70 °C. Expansion size was determined by chemiluminescence yielded by the binding of alkaline phosphatase-conjugated to anti-DIG antibody and CDP-Star substrate, according to the manufacturer’s instructions (Roche).

### 2.4. Small Pool-PCR

As opposed to the conventional PCR-Southern Blot, the SP-PCR technique uses small amounts of input DNA, allowing the study of single genomic equivalents, which are represented as single bands in the gel. SP-PCR was carried out using 300 pg of DNA in four replicates per sample, in order to study a representative repeat length distribution of the sample. We used the flanking primers DM-C and DM-DR as previously described [5,18], using a custom PCR Master Mix (Thermo Fisher Scientific; Waltham, MA, USA) supplemented with 69 mM 2-mercaptoethanol, and Taq polymerase Thermus aquaticus (Sigma-Aldrich; Gillingham, UK) at 1 unit per 10 µL. All reactions were supplemented with 5% DMSO and the annealing temperature was 63.5 °C. DNA fragments were resolved by electrophoresis on a 1% agarose gel, SB was hybridized using GE Healthcare Nylon Hybond N Membrane (Thermo Fisher Scientific; Waltham, MA, USA) as described [5,18], and autoradiographic images were scanned.

For LPCR-SB and SP-PCR, the CTG size of the progenitor allele and the mode allele (i.e., yielding the most intense band signal) of each patient were estimated by comparison against the molecular weight ladder, using GelAnalyzer 19.1 software. The length of the flanking CTG region of each PCR was subtracted for all the estimated CTG lengths.

### 2.5. Statistical Analysis

After checking that the data followed a normal distribution with the Kolmogorov–Smirnov test, we used a repeated-measures one-way analysis of variance (ANOVA) test for performing within-subject comparisons of the mean values of CTG expansion size of the progenitor allele and of the mode allele, respectively, obtained with the different methods. We also calculated the Pearson correlation between (i) the results yielded with the different methods, and (ii) the results obtained with each method and the age of disease onset, the Muscular Impairment Rating scale (MIRS), and the modified Rankin Scale (mRS), respectively, in the 15 DM1 patients. All statistical analyses were conducted using a statistical software package (SPSS 23), setting the significance level at α = 0.05.

## 3. Results

**Figure 1:**
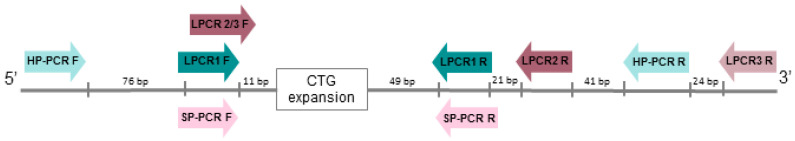
DMPK gene and location of the primer sets. Primers sets are indicated by the name of the technique and identified with a different background color. All of them were located outside the CTG expansion. LPCR2 and LPCR3 share the same forward primer. The distance (bp) between primers is also indicated. Abbreviations: F—forward; HPE-PCR—heat pulse extension-polymerase chain reaction; LPCR—long polymerase chain reaction; R—reverse; SP-PCR—small pool polymerase chain reaction.

A total of five primer sets were used (Figure 1), corresponding to the five different methods to measure CTG expansion size in our patients. All the primers were located outside the CTG repeat expansion. The name of each primer and its sequence are shown in Table 1. The length of each PCR product varied from 106 to 324 base pairs, depending on the primer set used (plus the number of CTG repeats for each patient) (Table 1). Thus, the differences in PCR amplification among the techniques were small.

**Figure 2:**
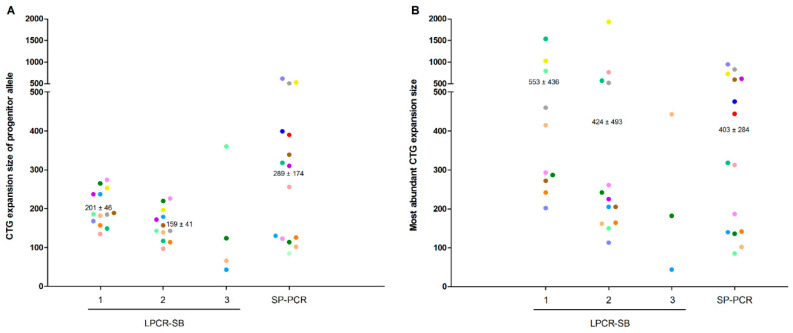
CTG expansion size of the progenitor allele (**A**) and the mode allele (**B**) for each technique. The progenitor allele was estimated by the lowest signal yielded after WT range for LPCR-SB, and by the most frequent lower band present in the samples for SP-PCR. Mode allele was estimated by the more intense signal yielded by LPCR and the most abundant band for SP-PCR. Data for heat pulse extension-polymerase chain reaction are not shown owing to the inconsistency of the results. All valid individual data within the detection limit (2000 CTGs) are shown(different color per patient). Normality was analyzed with the Kolmogorov–Smirnov test and we used a repeated-measures one-way analysis of variance (ANOVA) test for performing within-subject comparisons between methods. No significant group (or ‘method’) effect was found for the progenitor allele (*p* = 0.112) or the mode allele (*p* = 0.653). Mean and SD values are shown only for those methods included in the within-subject analyses (i.e., LPCR1, LPCR2 and SP-PCR). A significant Pearson correlation was found between the progenitor allele of LPCR1 and LPCR (indicated by an *symbol in the Figure, r = 0.983 [95% confidence interval (CI) 0.940 to 0.996], *p* < 0.0001). Y-axis scale is segmented from 500 CTGs to 2000 CTGs, representing 25% of the total length axis. Abbreviations: LPCR—long polymerase chain reaction; SP-PCR—small pool polymerase chain reaction.

Some technical difficulties were found with the HPE-PCR method. Although the technique worked in our hands, the results we obtained were inconsistent (Figure S1A) and thus, not comparable to previously published results [13]. Eight controls amplified smears that were similar to the ones found in patients—the status of control in our analysis is guaranteed, since prior to this analysis, we measured CTG alleles by sequencing. Thus, the cause of these unexpected smears is not apparent because they were unrelated to the CTG expansion in the study controls. As such, these data were excluded from statistical analyses. The results for all the methods, but HPE-PCR, are shown in Figure 2.

We also found problems with the LPCR-SB technique, which did not allow amplification of the CTG expansion in some patients (2 for LPCR1-SB and LPCR2-SB, respectively, and 11 for LPCR3-SB). Thus, based on the small amount of individual data points obtained with LPCR3-SB, we also excluded these data from statistical analyses. In LPCR, the amplified product in patients appeared sometimes as a high smear (Figure S1B), probably due to a mobility impairment in long amplifications of highly concentrated DNA (i.e., 100 ng in the final PCR reaction). Attending to the juvenile-classical phenotype of our patients, we established a detection limit of 2000 CTGs. In this context, three CTG sizes of the mode allele in LPCR1-SB were excluded from the study. None of the progenitor expansions surpassed the 2000 CTG-limit. SP-PCR amplified the CTG expansion of all the patients at the first attempt except for two of them—in whom, we had to repeat the amplification in order to correctly detect and quantify CTG expansion. Representative results of SP-PCR can be seen in Figure S1C.

**Figure 3:**
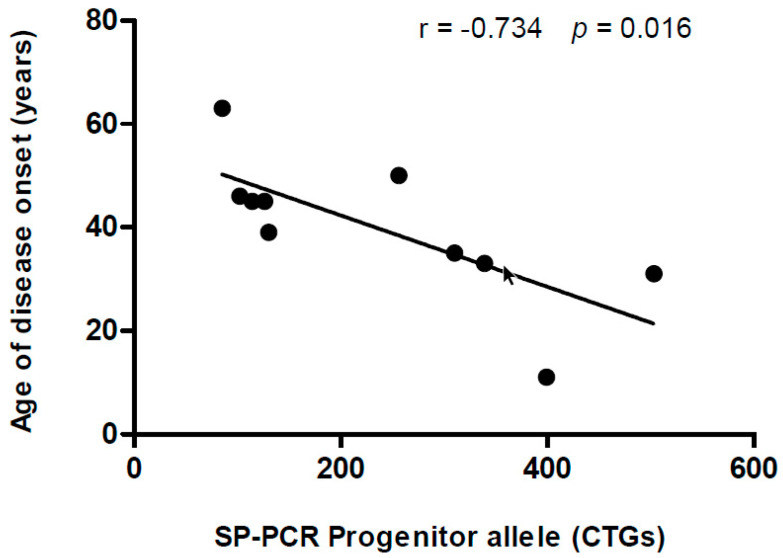
Correlation between the age of disease onset (years) and the CTG expansion size of the progenitor allele obtained through SP-PCR. The 95% confidence interval for the Pearson correlation coefficient was −0.933 to −0.1940 (r = −0.734, *p* = 0.016).

LPCRs yielded shorter progenitor alleles and higher mode alleles compared to SP-PCR (Figure 2). No significant group effect was found with the one-way repeated-measures ANOVA between the three techniques with analyzable data (i.e., LPCR1, LPCR2 and SP-PCR) for the within-subject comparison of CTG expansion size of the progenitor (*p* = 0.112) or mode allele (*p* = 0.653). A significant, strong correlation was found between LPCR1-SB and LPCR2-SB for CTG expansion size of the progenitor allele (r = 0.983 [95% confidence interval (CI) 0.940 to 0.996], *p* < 0.0001). However, no other significant correlation was found for the results obtained with LPCR1-SB, LPCR2 or SP-PCR, respectively (all *p* > 0.05). We further studied possible correlations with age of disease onset, MIRS and mRS scale. We found an inverse, significant correlation between CTG expansion size of the progenitor allele as determined by SP-PCR, and age of disease onset (Figure 3).

## 4. Discussion

Although no significant group effect was found with the one-way repeated-measures ANOVA between LPCR1/2 and SP-PCR, our results indicate that there is variability in the number of CTG repeats for a given patient depending on the CTG sizing method. HPE-PCR showed results that were difficult to interpret. Additionally, LPCR3 did not allow amplification of most of the DNAs in the patients. However, the fact that LPCR1 and LPCR-2 did yield some valid results suggests that LPCR-SB might be more sensitive to parameters such as the quality of the input DNA, which is not the case for the SP-PCR technique. SP-PCR was the only technique that enabled amplification of all DNAs from the patients and in fact, was the only one yielding a result that was correlated with an important phenotype trait of DM1—age of disease onset.

While a strong correlation was found between LPCR1-SB and LPCR2-SB for CTG expansion size of the progenitor allele, no other significant correlation was found. LPCR1/2-PCR yielded lower progenitor sizes and higher mode sizes than SP-PCR. LPCR-SB approaches usually show the expanded alleles as diffuse smears rather than discrete bands, due to the high input of DNA plus the somatic instability of the mutation [20]. This fact hinders differentiation of the progenitor allele size from possible contractions of the repeat. The number of PCR cycles may also affect the results—35 cycles are used in LPCR vs. 28 for SP-PCR—since a high number of cycles facilitates the amplification of shorter products, whereas longer products may be not favored. Moreover, the number of PCR cycles increases PCR slippage, tending to shorten the products. These phenomena could explain that these techniques yielded lower progenitor sizes than SP-PCR. By contrast, SP-PCR—which amplifies only small pools of input DNA—shows discrete bands that allow for a detailed analysis of the mutational spectrum and allele size distribution [18]. As such, this technique enables a better detection and estimation of the progenitor allele from post-contractions of the repeat. In three of the 15 patients, LPCR1-SB yielded some intense signals running high in the gel which, when measured, showed sizes above 2000 CTG repeats. Because the amount of input DNA is high (100 ng) in the different LPCR methods, the DNA mobility in the gel can be impaired, spreading out and yielding a signal that is higher than the actual CTG expansion size. Therefore, when using these LPCR-SB techniques, it would be necessary to set up a threshold for measuring CTG size in the detected smears.

It would be interesting to determine how novel, recently described technologies for CTG sizing [11,12] compare to the methods we assessed here. The sizing kit used by Leferink et al., was based on tripled repeat primed PCR, which is a robust and accurate technique to determine the presence of a CTG expanded allele [11]. However, the sizing of the repeat was limited in their study, set at 180 CTG repeats. In this regard, the most frequent DM1 form, the classical adult form, is usually associated with CTG repeats ranging from fifty to thousands. In fact, more than 70% of the samples in our study had more than 180 CTG repeats. Thus, although the kit reported in the Leferink et al. study would seem very useful for accurate DM1 diagnosis, it would not be suitable to size CTG expansion. In the study by Malbec et al., repeat sizing was performed with a lab-on-chip system that concentrates, separates, and detects DNA fragments in a very short time (actually, less than 5 min) from femtomolar concentrations of PCR-amplified DNAs [12]. Although this system appears as a good alternative to the sizing methods that we assessed, its accuracy would depend on the design of the primers used and the PCR amplification cycle. Furthermore, it would be also necessary to test some PCR designs in order to determine to what extent they are similar. Furthermore, since the chip presented in the Malbec et al. study can detect expansions up to 4Kb, it would have limitations to size samples from patients with congenital DM1. In fact, although their results were promising, only two DM1 blood samples were tested with the new technology, and as such, the interference of somatic mosaicism in CTG sizing remains to be analyzed.

Some studies have described that CTG repeat number can be a good indicator of disease onset [14]. In this regard, we further explored whether the different sizing results were related to the age of disease onset, finding a significant correlation for the SP-PCR method only. These results are overall in accordance with previous studies reporting a correlation between progenitor allele length measured by SP-PCR and both age of disease onset and clinical severity [10,21], although we found no correlation with MIRS or mRS. Progenitor allele length is the major modifier of age of disease onset, and as such, it is very important to use an accurate method for its determination. Concerning the lack of correlation with MIRS and mRS scales, CTG sizes in blood may be poor representatives of muscle status. Thus, future research in this field might study CTG in muscle cells.

## 5. Conclusions

Our study suggests that, besides the somatic mosaicism caused by CTG repeat instability and the inherent technical difficulties in assessing CTG expansion, there is overall heterogeneity among the different methods that are currently available, which makes it difficult to rely on them as valid predictors of disease phenotype. International agreement is needed to determine which is the most suitable methodology to characterize CTG expansion size in patients with DM1.

## Figures and Tables

**Table 1:** Techniques and primer sets used in this study.

Technique	bp of the Amplified Fragment (without CTG Expansion)	Primer Pair	Name	Sequence 5′—3′	Reference
HPE-PCR	324	F	DMKf	GCCAGTTCACAACCGCTCCGAGCGTGGGTC	Orpana et al. [13]
		R	DMKr	ACGCTCCCCAGAGCAGGGCGTCATGC	Orpana et al. [13]
LPCR1-SB	112	F	DM102	GAACGGGGCTCGAAGGGTCCTTGT	Brook et al. [1]
		R	DM101	CTTCCCAGGCCTGCAGTTTGCCCATCCA	Brook et al. [1]
LPCR2-SB	144	F	MDY1D	GCTCGAAGGGTCCTTGTAGCCG	Siciliano et al. [15]
		R	DM1REV	GTGCGTGGAGGATGGAAC	Radvansky et al. [17]
LPCR3-SB	262	F	MDY1D	GCTCGAAGGGTCCTTGTAGCCG	Siciliano et al. [15]
		R	SOMY4R	CGGGTTTGGCAAAAGCAAATTTCCCGA	Musova et al. [19]
SP-PCR	106	F	DM-C	AACGGGGCTCGAAGGGTCCT	Monckton et al. [5]; Gomes-Pereira et al. [18]
		R	DM-DR	CAGGCCTGCAGTTTGCCCATC	Monckton et al. [5]; Gomes-Pereira et al. [18]

Abbreviations: bp—base pairs; F—forward; HPE-PCR—heat pulse extension-polymerase chain reaction; LPCR—long polymerase chain reaction; R—reverse; SP-PCR—small pool polymerase chain reaction.

## Supplementary Materials

The following are available online at https://www.mdpi.com/2073-4425/11/7/757/s1. Figure S1: Representative results of the three different methods: HP-PCR, LPCR-SB and SP-PCR. (A) HP-PCR gel. Three patients and three controls are shown as representative results of HP-PCR. Controls showed similar signals to the ones found in patients. As such, these data were excluded from statistical analysis. (B) LPCR-SB gel showing the amplification results of the three primer sets: (1) DM102 and DM101 (LPCR1); (2) MDY1D and SOMY4R (LPCR2); and (3) MDY1D and DM1rev (LPCR3). Three patients and one control are shown. With this technique, we could not amplify the CTG expansion in some patients (two for LPCR1-SB and LPCR2-SB, respectively, and 11 for LPCR3-SB). The amplified product in patients appears as high smears, probably due to highly concentrated DNA (100 ng). (C). SP-PCR gel. Two patients and one control are shown, with four replicates per sample. Using small amounts of input DNA (300 pg), the technique allows us to study single genomic equivalents, which are amplified individually and further separated in the gel. Therefore, the gel shows discrete bands corresponding to individually CTG tracts of different lengths.
