# Supplementary figures and images for: The Need for Establishing a Universal CTG Sizing Method in Myotonic Dystrophy Type 1
Source: Genes (Basel). 2020 Jul 7;11(7):757. doi: 10.3390/genes11070757 (PMC7397178; doi:10.3390/genes11070757)

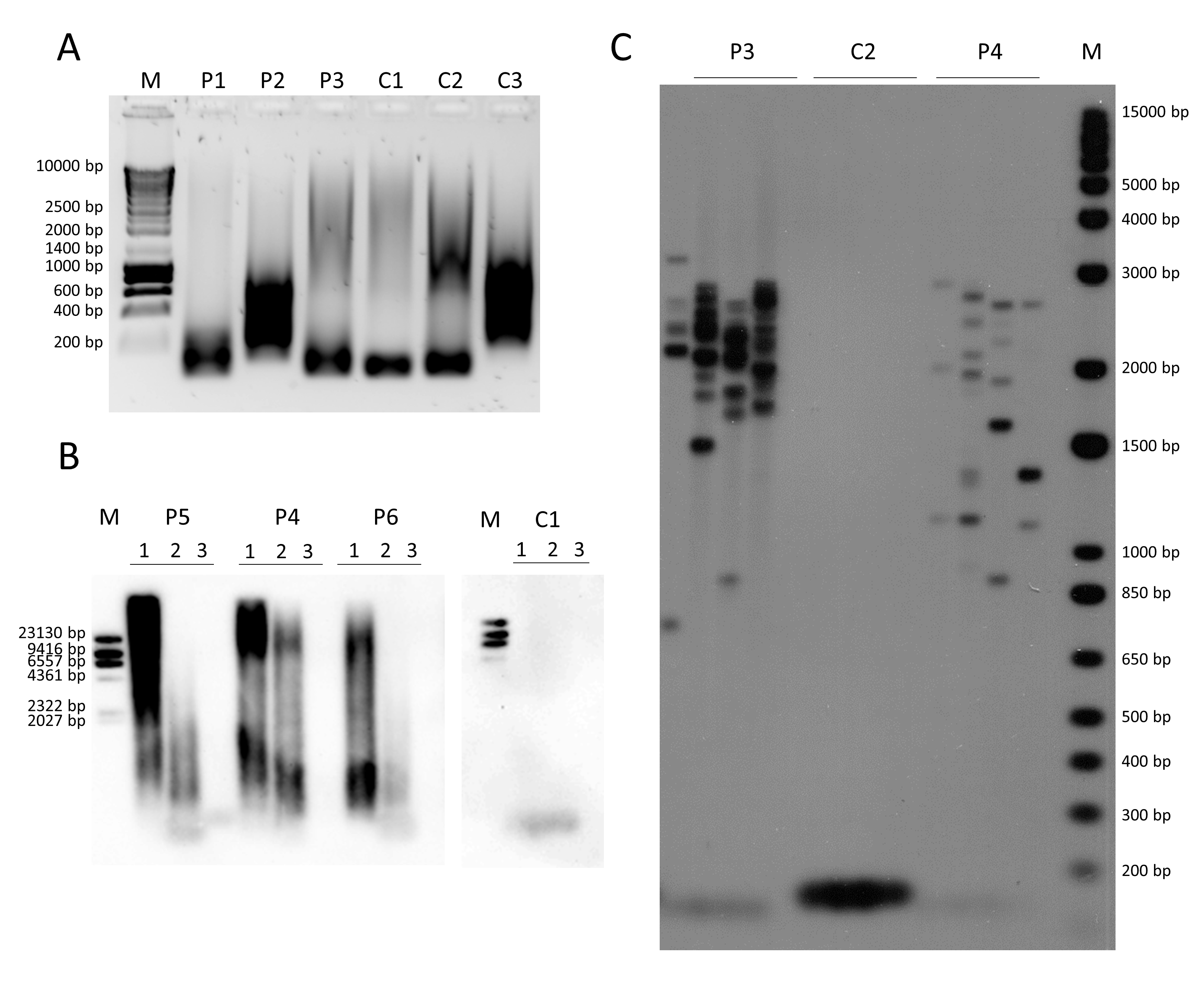

Supplement: Supplementary file 1 [file genes-11-00757-s001.zip › genes-832384-supplementary.tif]
